# Valorization of Grape Pomace and Berries as a New and Sustainable Dietary Supplement: Development, Characterization, and Antioxidant Activity Testing

**DOI:** 10.3390/nu14153065

**Published:** 2022-07-26

**Authors:** Adina Frum, Carmen Maximiliana Dobrea, Luca Liviu Rus, Lidia-Ioana Virchea, Claudiu Morgovan, Adriana Aurelia Chis, Anca Maria Arseniu, Anca Butuca, Felicia Gabriela Gligor, Laura Gratiela Vicas, Ovidiu Tita, Cecilia Georgescu

**Affiliations:** 1 Faculty of Medicine, “Lucian Blaga” University of Sibiu, 550169 Sibiu, Romania; adina.frum@ulbsibiu.ro (A.F.); lidia.virchea@ulbsibiu.ro (L.-I.V.); claudiu.morgovan@ulbsibiu.ro (C.M.); adriana.chis@ulbsibiu.ro (A.A.C.); anca.arseniu@ulbsibiu.ro (A.M.A.); anca.butuca@ulbsibiu.ro (A.B.); felicia.gligor@ulbsibiu.ro (F.G.G.); 2 Faculty of Medicine and Pharmacy, University of Oradea, 410028 Oradea, Romania; laura.vicas@gmail.com; 3 Faculty of Agriculture Science, Food Industry and Environmental Protection, “Lucian Blaga” University of Sibiu, 550012 Sibiu, Romania; ovidiu.tita@ulbsibiu.ro (O.T.); cecilia.georgescu@ulbsibiu.ro (C.G.)

**Keywords:** antioxidants, bilberry, by-products, food supplement, HPLC, phenolic compounds, polyphenols, pomace, red currant

## Abstract

Grape pomace and berries represent natural sources of phytochemicals that can increase the quality of life of consumers by contributing to the prevention of chronic diseases; thus, the development of a dietary supplement was necessary. The raw material (r.m.) used for the development of the dietary supplement consisted of dried and powdered bilberries (*Vaccinium myrtillus* L.), red currants (*Ribes rubrum* L.), and red fermented pomaces (*Vitis vinifera* L.) from Feteasca Neagra and Cabernet Sauvignon cultivars. The particle size distribution, powder flow, total phenolic content (TPC), HPLC-DAD phenolic profile assessment, and radical scavenging assay (RSA) were employed for the analysis of the raw material. After encapsulation, the average mass and uniformity of mass, the disintegration, and the uniformity of content for the obtained capsules were performed to obtain a high-quality dietary supplement. All the assays performed complied to the compendial requirements and the TPC was determined at 9.07 ± 0.25 mg gallic acid equivalents/g r.m. and RSA at 48.32 ± 0.74%. The highest quantities of phenolic compounds determined were 333.7 ± 0.50 µg/g r.m. for chlorogenic acid, followed by rutin, ferulic acid, and (+)-catechin with 198.9 ± 1.60 µg/g r.m., 179.8 ± 0.90 µg/g r.m. and 118.7 ± 0.75 µg/g r.m., respectively. The results of this study can be used for the manufacturing and assessing of pilot scale-up capsule batches and thinking of quality assurance, we recommend that the industrial batch extracts should be standardized in polyphenols, and the manufacturing process should be validated.

## 1. Introduction

Free radicals are unstable compounds that destroy biomolecules in the body, being responsible for the occurrence of oxidative stress. They have been associated with the development of cardiovascular disease, cancer, or other conditions [1,2]. By fighting free radicals, antioxidants protect the body from their harmful effects and can contribute to the prevention of such diseases [3,4]. 

For example, the consumption of natural polyphenols reduces the risk of myocardial infarctions and coronary heart disease due to their cardioprotective effect [5,6]. Flavonoid consumption prevents advanced stages of prostate cancer [7,8].

Metabolic syndrome includes a number of factors, such as chronic inflammation, hypertension, insulin resistance, high values of triglycerides, and low HDL (high-density lipoprotein) cholesterol levels that can lead to type 2 diabetes and cardiovascular disease. Atherosclerosis, a cause of cardiovascular disease, is associated with oxidative stress (OS) [6,9,10]. Diet plays an important role in the prevention of metabolic syndrome. The consumption of fruits and vegetables decreases the risk of metabolic syndrome and cardiovascular disease [6,11].

Berries contain phenolic compounds such as polyphenols, flavonoids, anthocyanins, flavonols, flavanols, stilbenes, and tannins [7,12,13]. Bilberries (*Vaccinium myrtillus* L. fruits) are considered to be valuable berries, as they capture a large amount of solar energy that they turn into compounds of importance for human health [3,14]. They have a nutritional value of approximately 399 kcal/ 100 g and contain mainly carbohydrates (94.5%), proteins (3%), lipids (1%), ash (1.5%), and also present bioactive compounds such as phenolic compounds, carotenoids, and vitamins. Bilberries are rich in anthocyanins, which are phenolic compounds responsible for fruit color [6,15]. The American Herbal Products Association recommends the administration of 160–480 mg of powdered extract divided into several doses per day or a daily dose of 20–60 g of dried bilberries [16,17]. Bilberry polyphenols have a role in controlling blood sugar in patients with type 2 diabetes, improving vision, inhibiting lipid peroxidation, and preventing the oxidative destruction of DNA [3].

Currants have an important energy value. They contain 9.2% carbohydrates, 4.2% fiber, 1.7% lipids, and 1.3% protein in the dry raw material. Red currants (*Ribes rubrum* L. fruits) are a rich source of vitamin C, polyphenols, and anthocyanins, which possess antioxidant properties [18,19]. Due to the rich content of polyphenols, currants (*Ribes* sp. L. fruits) are used in the management of diseases such as hypertension and other cardiovascular diseases, inflammation, osteoporosis, and cancer. They are important elements in the diet, due to their beneficial effects for the human body [7,20].

Both bilberries and currants are consumed fresh or processed in the form of juices, syrups, jams, but also in the form of extracts in food supplements or beverages [3,7,21,22]. The consumption of fresh berries brings a higher intake of polyphenols because some of them are lost through processing. However, processing offers the advantage of consuming bioactive compounds from berries throughout the year [3,23].

Over 67 million tons of grapes are produced annually, with grapes being the most cultivated fruit in the world [24,25]. Numerous losses occur as a result of food processing. Fortunately, the resulting waste from food processing is rich in bioactive compounds [26,27]. Many countries are investing in the extraction of bioactive compounds from by-products. The residue obtained when processing the grapes in order to make wine is called grape pomace and it represents approximately 20–25% of the mass of grapes subjected to processing [26,28,29]. It consists of peels, seeds, and stem fragments [26,30]. It also contains yeast results from the fermentation process [28]. In the past, pomace was considered a product without economic value, because it had no use [28], but now there is a tendency to obtain sustainable wines by the valorification of pomace [26]. Grape pomace is used to extract tartaric acid and obtain ethanol. The use of grape pomace as a fertilizer is disadvantageous because the polyphenolic compounds in its composition inhibit seed germination. Grape pomace has also been added to animal feed, but it has been observed that polymeric polyphenols inhibit cellulolytic and proteolytic enzymes and slow down digestion [24,31]. Grape pomace contains phenolic compounds (phenolic acids, anthocyanins, resveratrol, and procyanidins) [6,32] with important health benefits such as antioxidant, anti-inflammatory, antiproliferative and antimicrobial properties [26,28]. Grape pomace is used in the pharmaceutical, food, and cosmetics industries [26,33]. An interesting application of grape pomace is the production of ecocyanin, an anthocyanin used by the food industry as a food coloring [28]. 

An adequate diet could provide all the necessary nutrients for a healthy life, but because of lifestyles or other reasons people need to supplement their diet with the necessary nutrients by using dietary supplements [34]. They should be properly consumed when needed, because an inappropriate intake of dietary supplements can lead to adverse events [35,36]. 

The aim of this study is to develop a dietary supplement based on berries and a by-product of the vinification process that possesses antioxidant properties and can be used to supplement a faulty diet and to increase the quality of life by contributing to the prevention of chronic diseases. 

## 2. Materials and Methods

### 2.1. Chemicals

The standards of gallic acid (purity > 99%), ferulic acid (purity > 99%), syringic acid (purity > 95%), cinnamic acid (purity > 99%), caffeic acid (purity > 99%), (+)-catechin (purity > 98%), resveratrol (purity > 99%), chlorogenic acid (purity > 95%), quercetin (purity > 95%), rutin (purity > 94%), and methanol suitable for HPLC analysis (purity ≥ 99.9%) were purchased from Sigma-Aldrich (St. Louis, MO, USA). DPPH (2,2-Diphenyl-1-picrylhydrazyl) and Folin–Ciocalteu reagent, were purchased from Sigma-Aldrich (St. Louis, MO, USA) too and sodium acetate, glacial acetic acid, hydrochloric acid, and ethanol, all analytical grades, were purchased from the Chemical Company (Iasi, Romania). In all analyses, ultrapure water was used (conductivity 0.05 µS/cm).

### 2.2. Sample Preparation

The pulverulent raw material (r.m) used for the development of the dietary supplement consisted of bilberries (*Vaccinium myrtillus* L.), red currant (*Ribes rubrum* L.), and red fermented pomaces (*Vitis vinifera* L.) from Feteasca Neagra (FN) and Cabernet Sauvignon (CS) cultivars, added in equal parts (1:1:1). The berries were harvested from unpopulated areas of Sibiu County, Romania, and the pomaces were obtained from grapes harvested from a vineyard from Alba County, Romania. The berries and the pomace were dried in a Memmert UNB100 oven with continuous airflow at 40 °C and 20 °C, respectively, until they reached a constant mass and then they were ground on a domestic mill. The powder was calibrated using a 1.0 mm sieve. The mixing of the obtained powders was made by energic shaking for 10 min. The obtained powder was stored in amber glass containers at room temperature, away from sunlight until it was analyzed and encapsulated.

### 2.3. Raw Material Analysis

#### 2.3.1. Particle Size Distribution

Each sieve of the Retsch AS200 analytical sieve shaker was weighed and then the apparatus was assembled by putting the sieves in decreasing order of their sizes (710 µm, 224 µm, 125 µm, 90 µm, and 63 µm) and the collecting pan on the bottom. Exactly 100 g of raw material was weighed and put uniformly into the top sieve. After placing the cap and securing the assembled sieves the apparatus was set at 1.50 mm amplitude for 15 min. Each sieve was weighed, and the results were expressed as percentages (%).

Regarding the particle size distribution assessed by the sieve test, the powders are classified as coarse if through a 710 µm sieve passes a minimum 95% of the analyzed powder and through a 90 µm sieve passes a maximum 40% of it, moderately fine if through a 224 µm sieve passes a minimum 95% of the analyzed powder and through a 125 µm sieve passes a maximum 40% of it, fine if through a 125 µm sieve passes a minimum 95% of the analyzed powder and through a 90 µm sieve passes a maximum 40% of it, and very fine if through a 90 µm sieve passes a minimum 95% of the analyzed powder and through a 63 µm sieve passes a maximum 40% of it [37].

#### 2.3.2. Powder Flow

A total of 100 g of raw material was weighed and introduced into a 250 mL graduated cylinder. The unsettled apparent volume was registered (V_0_) and the cylinder was put on the Erweka SVM 102 tapped density tester. The apparatus was set at 10 beats, then 500 beats and then 1000 beats, until the registered volume remains the same. Then, the final tapped volume (V_f_) was registered. The Hausner ratio and the compressibility index was calculated by using the following formulas [37]:

$$\mathrm{Hausner}\;\mathrm{Ratio}=\frac{V_{0}}{V_{f}}$$

$$\mathrm{Compressibility}\;\mathrm{Index}=100\cdot \frac{V_{0}-V_{f}}{V_{f}}$$

The interpretation of the results was made by using the European Pharmacopoeia’s instructions, as follows: an excellent flow was characterized by a compressibility index between 1 and 10% and a Hausner ratio between 1.00 and 1.11, a good one by a compressibility index of 11–15% and a Hausner ratio of 1.12–1.18, a fair one by a compressibility index of 16–20% and a Hausner ratio of 1.19–1.25, a passable one by a compressibility index of 21–25% and a Hausner ratio of 1.26–1.34 and poor, very poor and very, very poor ones by compressibility indexes of 26–31, 32–37 and lower than 38, respectively and Hausner ratios of 1.35–1.45, 1.46–1.59 and below 1.60, respectively [37].

#### 2.3.3. Phytochemical Extraction 

A total of 500 mg of r.m. was weighed and added into a flask together with 10 mL of solvent (methanol: water: 0.12M hydrochloric acid = 70:29:1 (*V*/*V*/*V*)). The mixture was covered and put in an ultrasound bath at 40 °C for 30 min. After the time elapsed, the flask was cooled, and the mixture was filtered and brought to 10 mL into a volumetric flask with the same solvent. To determine the antioxidant activity of the r.m., the same extraction method was used by using methanol as the solvent [38]. 

#### 2.3.4. Phytochemical Analysis

##### Total Polyphenolic Content (TPC) Assay

A total of 0.4 mL sample solution, 1 mL of Folin–Ciocalteu reagent, 15 mL of water, and 2 mL of a 290 g/L Na_2_CO_3_ solution were added into a test tube, shaken for 10 min, and kept at 40 °C for 20 min in a water bath. After cooling, the extinction was recorded at λ = 760 nm by using a Thermo Scientific Evolution 300 Spectrophotometer [38,39]. The calibration curve was linear for the range of 0.9–4.5 µg gallic acid/mL, the equation was y = 0.258x + 0.022 (R^2^ = 0.998) where y = extinction at λ = 760 nm and x = concentration expressed as µg gallic acid/ mL, and the results were expressed as mg gallic acid equivalents (GAE)/g r.m. and the analysis was performed in triplicate.

##### HPLC-DAD Phenolic Profile Assessment

The HPLC-DAD method for the qualitative and quantitative assessment of the ten phenolic compounds analyzed was obtained by using methods already applied on plants and food supplements [38,40,41]. The analysis was carried out on an Agilent technologies 1200 series HPLC system equipped with a degasser, a quaternary pump, a diode array detector, a thermostatted column compartment, and an autosampler. The column that was used was Zorbax Eclipse Plus C18 (250 mm × 4.6 mm i.d. × 5 µm), which was kept at a temperature of 25 °C. The elution was performed by using three mobile phases in gradient (mobile phase A = purified water, mobile phase B = methanol, and mobile phase C = purified water: acetic acid = 96:4 *V*/*V*). The gradient program was used with 15% B and 85% C at 0 min, 75% A and 25% B at 15 min, 15% A and 85% B at 20 min, 40% A and 60% B at 40 min, 5% A and 95% B at 45 min, 5% A and 95% B at 55 min, 85% A and 15% B at 60 min, and 85% A and 15% B at 70 min. The flow rate program was 0.5 mL/min for the first 15 min and from minute 15 to minute 70, 0.8 mL/min and the injection volume was 5 µL. The detection was performed at 280 nm for gallic acid, (+)-catechin, syringic acid, and cinnamic acid, 303 nm for resveratrol, 330 nm for chlorogenic acid, caffeic acid, and ferulic acid, and 360 nm for rutin and quercetin. The results were expressed as µg phenolic compound/g r.m. and the analysis was performed in triplicate.

##### Antioxidant Activity Assay

The antioxidant activity assay was performed by using the 2,2-diphenyl-1-picrylhydrazyl free radical scavenging assay (RSA). A stock solution of 25 µg/mL DPPH in methanol was prepared and kept at a low temperature and in the dark for 2 h before use. A total of 970 µL of DPPH stock solution was added into 30 µL of sample solution. The absorbance was recorded at 517 nm, using a Thermo Scientific Evolution 300 Spectrophotometer. The results were expressed as percentages and the analysis was performed in triplicate [42,43]. The calibration curve was linear for the range of DPPH concentrations of 0.25–250 µg/mL. The equation based on the calibration curve was y = 0.0411x + 0.0349 (R^2^ = 0.999), where y = extinction at λ = 593 nm and x = concentration expressed as µg DPPH/mL. The DPPH radical scavenging activity was determined by using the following formula:

$$\mathrm{RSA}\;\left(\%\right)=\frac{C_{0}-C_{1}}{C_{0}}\cdot 100$$

where: RSA = DPPH radical scavenging activity (%), C_0_ = concentration of the DPPH stock solution (µg/mL), and C_1_ = DPPH concentration in the sample (µg/mL).

### 2.4. Encapsulation

A total of 35 g of r.m. was filled into hard capsules size 00 and then the capsules were closed by locking the caps by using a pharmaceutical grade manual capsule filler.

### 2.5. Analysis of Capsules

#### 2.5.1. Average Mass and Uniformity of Mass

Twenty full capsules were individually weighed and then they were opened without losing any part of the shell and the content was removed as completely as possible. The shell was weighed and the difference between the full capsule and the shell represents the weight of the capsule’s content. The average mass was calculated by using the following formula:

$$\mathrm{Average}\;\mathrm{mass}\;\left(\mathrm{mg}/\mathrm{capsule}\right)=\frac{\sum_{i=1}^{n}M_{i}}{n}$$

where: M_i_ = the individual weighing of each capsule and n = the number of weight capsules [37].

In order for the capsules to comply to the requirements of the European Pharmacopoeia, the 10th edition, the average mass of the full capsules has to be 820.0 mg/capsule ± 7.5% (758.5–881.5 mg/capsule), the average mass of the content of the capsules has to be 700.0 mg/capsule± 7.5% (647.5–752.5 mg/capsule), and at least 18 capsules must have a deviation of at most ± 7.5% from the average mass and no more than 2 capsules can have a deviation of at most ± 15% from the average mass.

#### 2.5.2. Disintegration

Six capsules were placed each in one of the 6 tubes of the basket of an Erweka ZT 72 Disintegratin Tester and a sensor disk was placed on top of each capsule. The determination was employed by using water as the immersion fluid at 37 ± 2 °C. The full disintegration of the capsules was observed. The test is considered finished after all the analyzed capsules are disintegrated. For the capsules to comply to the requirements of the European Pharmacopoeia, the 10th edition, the disintegration time must be less than 30 min for all the capsules. If 1 or 2 capsules are not completely disintegrated, the test will be repeated for 12 capsules. The test is compliant if 16 of 18 capsules are disintegrated in less than 30 min [37].

#### 2.5.3. Uniformity of Content

To determine the uniformity of the content of phytochemicals within the obtained capsules, the same extraction and analysis as for the r.m. were performed as presented in Section 2.3.3 and Section 2.3.4. The powder to be analyzed was the content of 20 capsules that was mixed properly, and the results were expressed as content of phytochemical per capsule [37].

## 3. Results

### 3.1. Raw Material analysis

#### 3.1.1. Particle Size Distribution

From the analysis of the results obtained, it can be observed that the analyzed r.m. is a coarse powder, because 95.36% of the added r.m. passed through the 710 µm sieve and 0.37% through the 224 µm sieve (Table 1).

#### 3.1.2. Powder Flow

The powder flow was good according to the obtained results (Table 2) that were interpreted by using Section 2.3.2.

This parameter shows the flowability of the r.m. and assesses the possibility of this powder to be encapsulated without any difficulties even if no excipients that improve the flowability were to be added. 

#### 3.1.3. Phytochemical Analysis

**Figure 1:**
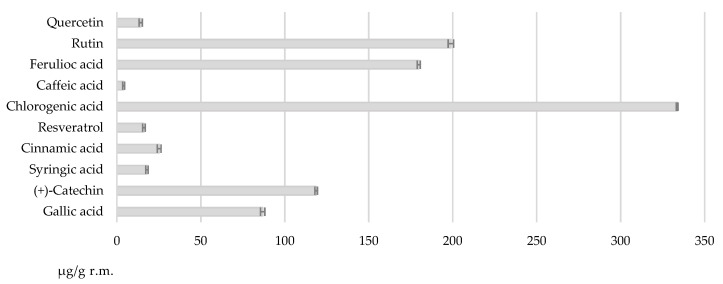
Quantification of phenolic compounds assessed for the r.m.

The analyzed r.m. presented 9.07 ± 0.25 mg GAE/g r.m. TPC and 48.32 ± 0.74% RSA. The analyzed phenolic compounds can be observed in Figure 1. The results were expressed as mean ± standard deviation (SD) for three determinations (n = 3).

The highest quantity of phenolic compound determined was 333.7 ± 0.50 µg/g r.m. for chlorogenic acid. Values between 100 and 200 µg/g r.m. had rutin, ferulic acid, and (+)-catechin with 198.9 ± 1.60 µg/g r.m., 179.8 ± 0.90 µg/g r.m., and 118.7 ± 0.75 µg/g r.m., respectively. The values obtained for gallic acid, cinnamic acid, resveratrol, syringic acid, quercetin, and caffeic acid were beneath 100 µg/g r.m. (Figure 1).

The percentages calculated for the phenolic compounds analyzed were: 33.53% for chlorogenic acid, 19.98% for rutin, 18.06% for ferulic acid, 11.93 for (+)-catechin, 8.72% for gallic acid, 2.53% for cinnamic acid, 1.62% for resveratrol, 1.80% for syringic acid, 1.43% for quercetin, and 0.40% for caffeic acid.

### 3.2. Analysis of Capsules

#### 3.2.1. Average Mass and Uniformity of Mass

Given that all the analyzed capsules fell into the ±7.5% deviation regarding the average mass and uniformity of mass (Table 3), it can be concluded that the analyzed capsules comply to the requirements of the European Pharmacopoeia. 

#### 3.2.2. Disintegration

The disintegration of the six capsules analyzed was performed in under 3 min (Table 4), thus the requirements of the European Pharmacopoeia regarding this analysis were met.

#### 3.2.3. Uniformity of Content

**Figure 2:**
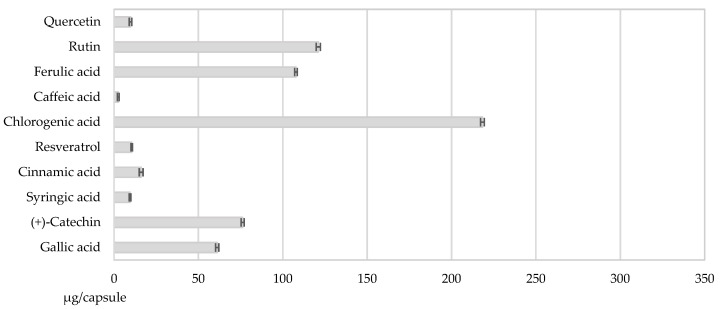
The uniformity of content in phenolic compounds of the capsules analyzed.

This test is performed to assess the uniformity of phytochemicals within the obtained capsules. The capsules analyzed presented 6.60 ± 0.44 mg GAE/capsule TPC. The highest quantity of phytochemical obtained was for chlorogenic acid, which had 218.33 ± 1.12 µg/capsule, followed by rutin that presented an amount of 121.03 ± 1.22 µg/capsule and then by ferulic acid with a quantity of 107.75 ± 0.79 µg/capsule. The rest of the analyzed phenolic compounds presented values that were below 100 µg/capsule (Figure 2). The results were expressed as mean ± SD for three determinations (n = 3).

The percentages of the phenolic compounds analyzed per capsule were as follows: 34.51% chlorogenic acid, 19.13% rutin, 17.03% ferulic acid, 12.04% (+)-catechin, 9.66% gallic acid, 2.54% cinnamic acid, 1.65% resveratrol, 1.50% syringic acid, 1.55% quercetin, and 0.40% caffeic acid.

## 4. Discussion

Grape pomace is represented by peels, seeds, and stem fragments of the grapes used for industrial processes, mainly the vinification process [28,44,45]. It can be considered a burden for the environment, and it causes financial losses if it is not valorized [46,47]. Iqbal et al. (2021) and Antonic et al. (2020) state that grape pomace can be considered a source of dietary fiber, unsaturated fatty acids, mono- and polysaccharides, and a significant source of polyphenols that can be used as functional foods or ingredients that add value to food products [46,47], thus the valorization of this by-product can be beneficial to humanity and the environment as well. Ferreira et al. (2022) states that grape pomace can be used in the cosmetic industry as skin hydration products, in the food industry as functional foods or additives, in agriculture as fertilizers or animal feed, and in the pharmaceutical industry as dietary supplements or drug administration systems [48]. 

The tests performed on the raw material and the obtained capsules are mandatory for the development of any proper dietary supplement. These tests provide information regarding the efficiency of the proposed vegetal material as a dietary supplement [49,50].

Studies conducted before for the assessment of phenolic compounds in bilberries, red currants, and fermented red pomaces from the CS and FN cultivar reveal higher quantities than 400 µg (+)-catechin/g vegetal product dry weight (d.w.) for FN and CS pomaces and red currants. Rutin had the greatest amount in bilberries (greater than 350 µg/g d.w.) followed by CS pomace with a concentration greater than 100 µg/g d.w. [38,51]. Bilberries had amounts greater than 900 µg/g d.w. for chlorogenic acid and greater than 350 µg/g d.w. for ferulic acid and rutin. Quantities greater than 300 µg/g d.w. of syringic acid were determined for red currants and greater than 150 µg/g d.w. of gallic acid for bilberries and red currants. Caffeic acid and chlorogenic acid were not detected in both the analyzed pomaces, and quercetin, caffeic acid, and resveratrol in red currants [38,51]. The mixture of these vegetal products provides a wider range of phenolic compounds in higher quantities than each compound would have alone, thus providing a better intake of phenolic compounds to consumers. In most of the cases, antioxidants from multiple sources have proven superior to an individual compound [52].

The pathogenesis of chronic disease has often been related with OS [53,54,55]. Several molecules of major interest such as DNA, proteins, and lipids can be damaged by reactive oxygen species (ROS) and reactive nitrogen species (RNS) [56,57]. The mechanism of action of antioxidants has been extensively studied, and their beneficial effect against DNA methylation [58], lipid peroxidation [59], and protein damage [60] has been reported. Polyphenols and other antioxidants have successfully influenced the health status of patients diagnosed with a variety of chronic conditions, such as cardiovascular disease [61,62], cancer [63,64], endometriosis [65], osteoarthritis [66,67], Parkinson’s [68], and Alzheimer’s disease [69,70]. In most of the cases, antioxidants from multiple sources have proven superior to an individual compound [52,71].

In vitro, grape pomace displays high antioxidant potential [72], lowers PGE2 levels, and downregulates COX-2 gene expression [73]. Furthermore, grape pomace polyphenols have proven their efficacy in vivo, by delivering antiglycation agents and preventing the formation of toxic glycation end-products [74].

Even though polyphenols present antioxidant activity, with numerous benefits in preserving human health, the pro-oxidant effect of these compounds must be recognized [75,76,77]. When consumed in low or moderate concentrations, pro-oxidants can be considered as weapons for the defense system of the organism and if consumed in high doses, they have toxic potential by causing OS. On the other hand, the ability of pro-oxidants to produce cell apoptosis can be exploited in cancer therapy [76,77,78]. Thus, polyphenol-rich dietary supplements could offer health benefits to consumers when administered in appropriate doses [79].

During the technological development of our dietary supplement, we followed the high quality standard guidance of good manufacturing practices for pharmaceuticals, because two thirds of small molecule-active pharmaceutical ingredients are of natural origin [80]. The quality of the finished product is a sum of all the factors contributing to its manufacturing. Through proper documentation, traceability ensures high quality levels for each stage of the manufacturing process, starting with the raw materials and continuing with the manufacturing process and equipment, intermediary or bulk products, and analytical methods and equipment [81].

The pharmaco-technical methods used for the evaluation of the pharmaceutical form and raw materials were in accordance with the European Pharmacopoeia. This compendium standardizes a mechanical method for particle size distribution estimation by analytical sieving and recommends it for powders where the majority of the particles are larger than approximately 75 μm [37]. Modern methods for assessing the particle size of fine powder use particle size analyzers or light scattering spectrophotometers [82].

The dimensions of particles play a crucial role in the quality of the finished product on multiple levels. First, they affect the production process by determining flow properties [83]. Second, they impact disintegration and dissolution, influencing both bioavailability and pharmacodynamics [84]. The decision to continue the study without further reduction of raw material to nano-size particles was based on a series of factors: (i) the avoidance of a supplementary process stage; (ii) the negative impact fines have on rheological properties during manufacturing [83]; (iii) the cost efficiency of the formulation by avoiding unnecessary excipients for enhancing the flowability.

The results of this study can be rapidly employed to manufacturing and assessing pilot scale-up capsule batches. Having in mind quality assurance, it is recommended that the industrial batch extracts should be standardized in polyphenols, and the manufacturing process should be validated [85]. Such a dietary supplement would combine several benefits: a high acceptance by the population, a precise dosage and a stability of oral solid dosage, a production requiring regular manufacturing equipment, and compendial quality control methods. A good adherence to polyphenol-rich supplements ensures the bioavailability of these active compounds and constitutes the first stage towards the onset of their pharmacodynamic potential [86]. 

## 5. Conclusions

The valorization of pomace, a by-product of the vinification process, is of utmost importance for the preservation of the environment and for financial purposes. The obtained dietary supplement combines bilberries, red currants, and fermented red pomaces obtained from two cultivars, it complies to quality requirements, and it provides a wide range of phenolic compounds, thus providing an increase in the quality of life of the consumers by contributing to the prevention of chronic diseases. 

Although polyphenol-rich dietary supplements could possess beneficial properties for human health, tests regarding their bioavailability, in vivo studies, and clinical trials that provide evidence of their therapeutic potential and toxicity remain to be assessed.

## Figures and Tables

**Table 1:** R.m. particle size distribution.

Sieve (µm)	Weight (g)	Weight (%)
710	4.25	4.27
224	94.93	95.36
125	0.37	0.37
90	0.00	0.00
63	0.00	0.00

**Table 2:** R.m powder flow assessment.

Number of Beats	Volume (mL)	Compressibility Index	Hausner Ratio
0	208	14.71%	1.17
10	184		
500	174		
1000	174		

**Table 3:** Average mass and uniformity of mass assessment.

Capsule No.	Full Capsule (mg)	Content (mg)
1	820	703
2	827	707
3	817	701
4	815	700
5	823	702
6	820	701
7	835	713
8	827	712
9	822	702
10	827	707
11	831	713
12	817	698
13	826	707
14	830	709
15	815	700
16	826	708
17	819	699
18	824	707
19	815	696
20	823	703
Average	822.95	704.4

**Table 4:** The time elapsed for the disintegration of capsules.

Basket No.	1	2	3	4	5	6
**Time (Minutes:Seconds)**	2:10	2:03	1:20	2:13	2:35	1:59

## Data Availability

Not applicable.

## Abbreviations

CS	Cabernet Sauvignon
d.w.	dry weight
DPPH	2,2-Diphenyl-1-picrylhydrazyl
FN	Feteasca Neagra
GAE	gallic acid equivalents
OS	oxidative stress
r.m.	raw material
RNS	reactive nitrogen species
ROS	reactive oxygen species
RSA	radical scavenging assay
SD	standard deviation
TPC	total phenolic content
V_0_	apparent volume
V_f_	final volume
